# Clinical analysis in patients with *SPG11* hereditary spastic paraplegia

**DOI:** 10.3389/fneur.2023.1198728

**Published:** 2023-06-15

**Authors:** You-Ri Kang, Tai-Seung Nam, Jae-Myung Kim, Kyung Wook Kang, Seong-Min Choi, Seung-Han Lee, Byeong C. Kim, Myeong-Kyu Kim

**Affiliations:** ^1^Department of Neurology, Chonnam National University Hospital, Gwangju, Republic of Korea; ^2^Department of Neurology, Chonnam National University Medical School, Gwangju, Republic of Korea

**Keywords:** *SPG11*, spastic paraplegia, corpus callosum, magnetic resonance imaging, evoked potential (EP), intellectual disabilities

## Abstract

**Background:**

To analyze the clinical phenotype of hereditary spastic paraplegia (HSP) caused by *SPG11* mutations (SPG11-HSP).

**Methods:**

Among the 17 patients with sporadic HSP who performed whole exome sequencing analysis, six were diagnosed with SPG11-HSP. The clinical and radiologic findings and the results of the electrodiagnostic and neuropsychologic tests were reviewed retrospectively.

**Results:**

The median age at onset was 16.5 years (range, 13–38 years). Progressive spastic paraparesis was a core feature, and the median spastic paraplegia rating scale score was 24/52 (range, 16–31 points). Additional major symptoms were pseudobulbar dysarthria, intellectual disability, bladder dysfunction, and being overweight. Minor symptoms included upper limbs rigidity and sensory axonopathy. The median body mass index was 26.2 kg/m^2^ (range, 25.2–32.3 kg/m^2^). The thin corpus callosum (TCC) was predominant at the rostral body or anterior midbody, and the ears of the lynx sign was seen in all. The follow-up MRI showed the worsening of periventricular white matter (PVWM) signal abnormalities with ventricular widening or the extension of the TCC. Motor evoked potentials (MEP) to the lower limbs showed an absent central motor conduction time (CMCT) in all subjects. The upper limb CMCT was initially absent in three subjects, although it became abnormal in all at the follow-up. The mini-mental state examination median score was 27/30 (range, 26–28) with selective impairment of the attention/calculation domain. The median score of the full-scale intelligence quotient was 48 (range, 42–72) on the Wechsler Adult Intelligence Scale test.

**Conclusion:**

Attention/calculation deficits and being overweight as well as pseudobulbar dysarthria were common additional symptoms in patients with SPG11-HSP. The rostral body and anterior midbody of the corpus callosum were preferentially thinned, especially in the early stage of the disease. The TCC, PVWM signal changes, and MEP abnormality worsened as the disease progressed.

## Introduction

Hereditary spastic paraplegia (HSP) is a group of inherited neurological disorders characterized by progressive spastic weakness of the lower limbs due to degeneration of the corticospinal tracts (1). HSP is clinically divided into two subtypes: (a) pure or uncomplicated HSP, which is characterized by difficulty walking due to progressive spastic paraplegia of insidious onset; (b) complicated HSP, which is associated with additional neurologic symptoms or medical conditions, including intellectual disability, dementia, ataxia, extrapyramidal disorders, optic atrophy, peripheral neuropathy, amyotrophy, or epilepsy (1–3). The biallelic mutations in the *SPG11* gene encoding the spatacsin protein can lead to complicated HSP (SPG11-HSP). SPG11-HSP is a rare disease with an incidence of 0.35 per 100,000 people and is known to account for 19%–31% of autosomal recessive HSP (4–6). SPG11-HSP may clinically manifest as various neurological symptoms or signs associated with the central and peripheral nervous involvement (5, 7). The thin corpus callosum (TCC) is known as a radiologic hallmark of SPG11-HSP (5, 8). However, TCC cannot be pathognomonic, considering that it is also observed in HSP with other genotypes (8, 9), and several cases of SPG11-HSP without TCC have been reported (10). Thus, a more comprehensive study is needed to understand this rare disease with phenotypic diversity. This study aimed to comprehensively analyze the phenotype of SPG11-HSP, including clinical, radiological, electrodiagnostic, and neuropsychologic features.

## Materials and methods

### Subjects

We searched for patients undergoing follow-ups at the outpatient neurology clinic after being diagnosed with sporadic HSP. Whole exome sequencing (WES) was performed to identify the causative gene in 17 patients, of which 5 were classified into pure HSP and 12 were into complicated HSP. Six patients with complicated HSP were considered as having SPG11-HSP. In this study, the diagnosis of SPG11-HSP is defined as the presence of slowly progressive spastic paraparesis, TCC on MRI scan, identification of pathogenic mutations in the *SPG11* gene, but no significant variants identified in more than 80 other HSP-related genes. We retrospectively reviewed the medical records of six subjects, including two (subject number (SN)-5 and SN-6) identical twins. Five known pathogenic and two novel variants in the *SPG11* gene were identified (Table 1). The twins carried an additional pathogenic heterozygous c.950G > A/p.Arg317Gln variant in the *CLCN1* gene (11, 12). A detailed description of the WES analysis, the flowchart of patient selection, and information on all genetic variants found in the *SPG11* gene in this study are provided in the Supplementary materials. This project was approved by the institutional review board of the Chonnam National University Hospital (CNUH-2020-018).

**Table 1:** Variants identified in the *SPG11* gene.

Patient	Exon	c.DNA change	Amino acid change	Zygosity	ACMG-AMP criteria^a^	ACMG classification	dbSNP No.
SN-1	Intron	c.3291+1G>T	NA	Heterozygous	PVS1 + PM2 + PP3-5	Pathogenic	rs312262753
	25	c.4307_4308delAA	p.Gln1436Argfs	Heterozygous	PVS1 + PM2 + PP3-5	Pathogenic	rs312262759
SN-2	Intron	c.3291+1G>T	NA	Heterozygous	PVS1 + PM2 + PP3-5	Pathogenic	rs312262753
SN-3	1	c.200_203delCTTT	p.Ser67fs	Heterozygous	PVS1 + PM2 + PP3/4	Pathogenic	NA
	39	c.7010T>G	p.Val2337Gly	Heterozygous	PM2 + PP3/4	VUS	NA
SN-4	16	c.2987_2989delGTT	p.Cys996del	Heterozygous	PVS1 + PM2/4 + PP3-5	Pathogenic	NA
SN-5	11	c.2163dupT	p.Ile722Tyrfs	Heterozygous	PVS1 + PM2 + PP3-5	Pathogenic	rs312262738
	30	c.5410_5411delTG	p.Cys1804Profs	Heterozygous	PVS1 + PM2 + PP3-5	Pathogenic	rs312262766
SN-6	11	c.2163dupT	p.Ile722Tyrfs	Heterozygous	PVS1 + PM2 + PP3-5	Pathogenic	rs312262738
	30	c.5410_5411delTG	p.Cys1804Profs	Heterozygous	PVS1 + PM2 + PP3-5	Pathogenic	rs312262766

SN, subject number; ACMG, the American College of Medical Genetics and Genomics; AMP, the Association for Molecular Pathology; VUS, variant of uncertain significance; NA, not available; SN-5 and SN-6 are identical twins.

a Evidence of pathogenicity very strong (PVS), strong (PS), moderate (PM), supporting (PP).

### Methods

The clinical characteristics were established by obtaining the following: (1) *Clinical findings* included demographic information, including body mass index (BMI), subjective symptoms, and neurological examinations. The spastic paraplegia rating scale (SPRS) was measured (13). (2) *Radiological findings* included the TCC and periventricular white matter (PVWM) signal abnormalities. Midsagittal T1-weighted images were selected to assess the affected regions of the corpus callosum, which was divided into seven subdivisions according to Witelson’s guidelines: region 1 (rostrum), region 2 (genu), region 3 (rostral body), region 4 (anterior midbody), region 5 (posterior midbody), region 6 (isthmus), and region 7 (splenium) (14, 15). PVWM signal abnormalities were evaluated on an axial fluid-attenuated inversion recovery (FLAIR) image, and ventricular widening was investigated. (3) *Electrodiagnostic findings* included the results from the motor evoked potentials (MEP) and somatosensory evoked potentials (SEP), the nerve conduction study (NCS), and electromyography (EMG). Central motor conduction time (CMCT) was measured by subtracting the peripheral motor conduction time (PMCT) from the onset latencies of the MEP. PMCT was calculated by adding F-wave latency and M-wave latency measured in the posterior tibial and ulnar nerves and then subtracting 1 msec, reflecting the turnaround time at the anterior horn cell and dividing by two. Thus, the formula used for calculating the CMCT is as follows: CMCT (msec) = MEP – (F-latency + M-latency – 1)/2 (16). (4) *Neuropsychologic findings* included the results of the Korean version of the mini-mental state examination (K-MMSE) and the Korean Wechsler Adult Intelligence Scale (K-WAIS) test for the evaluation of the intelligence quotient (IQ).

## Results

The subjects were between 17 and 41 years old. All subjects had no family history of neurodegenerative diseases. Three subjects (SN-1, SN-2, and SN-3) had a history of intellectual disability. Five subjects were educated for over 9 years, including the highly educated one (SN-4) who graduated from university. The median BMI was 26.2 kg/m^2^ (range, 25.2–32.3 kg/m^2^). All subjects presented with slowly progressive spasticity in the lower limbs. The median age at the onset of gait disturbance was 16.5 years (range, 13–38 years); it developed in late adolescence in five subjects, while the other (SN-4) experienced it in her late thirties. Five of the six subjects could walk independently at the initial examination. All became dependent on ambulatory assistive devices (AAD) with time, and the median duration from onset to use of AAD was 5 years. The median SPRS score was 24/52 (range, 16–31 points). Learning difficulties and urinary or bowel incontinence were common. One subject (SN-1) complained of rigidity or stiffness in the upper limbs, another (SN-4) had paresthesia in the lower limbs, and the twins (SN-5 and SN-6) had experienced painful muscle stiffness in their calves since their early teens. Neurological examinations generally revealed pseudobulbar dysarthria, positive Babinski sign and ankle clonus on both sides, and Achilles tendon contracture. Calf muscle hypertrophy was seen in the twins. The clinical manifestations are summarized in Table 2.

**Table 2:** Clinical findings of the enrolled patients in this study.

Subjects	SN-1	SN-2	SN-3	SN-4	SN-5	SN-6
Gender	F	M	M	F	M	M
Age at onset (years)	16	13	13	38	17	17
Age at examination (years)	18	17	22	41	21	21
Follow-up period (years)	13	3	2.5	4	8	8
Family history	−	−	−	−	+	+
BMI (kg/m^2^)	28.4	26.5	25.8	32.3	25.2	25.8
Ambulatory status						
At examination	Self	Self	Crutch	Self	Self	Self
Onset to AAD (years)	4	6	UNK	4	5	5
Clinical manifestation						
Slurred speech	+	+	+	+	+	+
Learning difficulties	+	+	+	±	+	+
Urinary incontinence	+	+	+	+	+	+
Fecal incontinence	+	+	+	+	−	−
Myalgia	−	−	−	−	+	+
Neurologic examination						
SPRS score	18	24	31	16	24	25
Lower limb spasticity	+	+	+	+	+	+
Achilles contracture	+	+	+	+	+	+
Pseudobulbar dysarthria	+	+	+	+	+	+
Deep tendon reflex						
Upper limbs	2+/2+	2+/2+	2+/2+	2+/2+	2+/2+	2+/2+
Lower limbs	4+/4+	4+/4+	4+/4+	4+/4+	4+/4+	4+/4+
Babinski sign	+/+	+/+	+/+	+/+	+/+	+/+
Ankle clonus	+/+	+/+	+/+	+/+	+/+	+/+
Hoffman’s sign	−/−	−/−	−/−	−/−	−/−	−/−
Upper limb rigidity	+	−	−	−	−	−
Extrapyramidal sign	−	−	−	−	−	−
Sensory loss	−	−	−	+	−	−
Calf muscle hypertrophy	−	−	−	−	+	+
Muscle atrophy	−	−	−	−	−	−

SN, subject number; F, female; M, male; +, present; −, absent; BMI, body mass index; AAD, ambulatory assistive devices; UNK, unknown; SPRS, spastic paraplegia rating scale.

**Figure 1:**
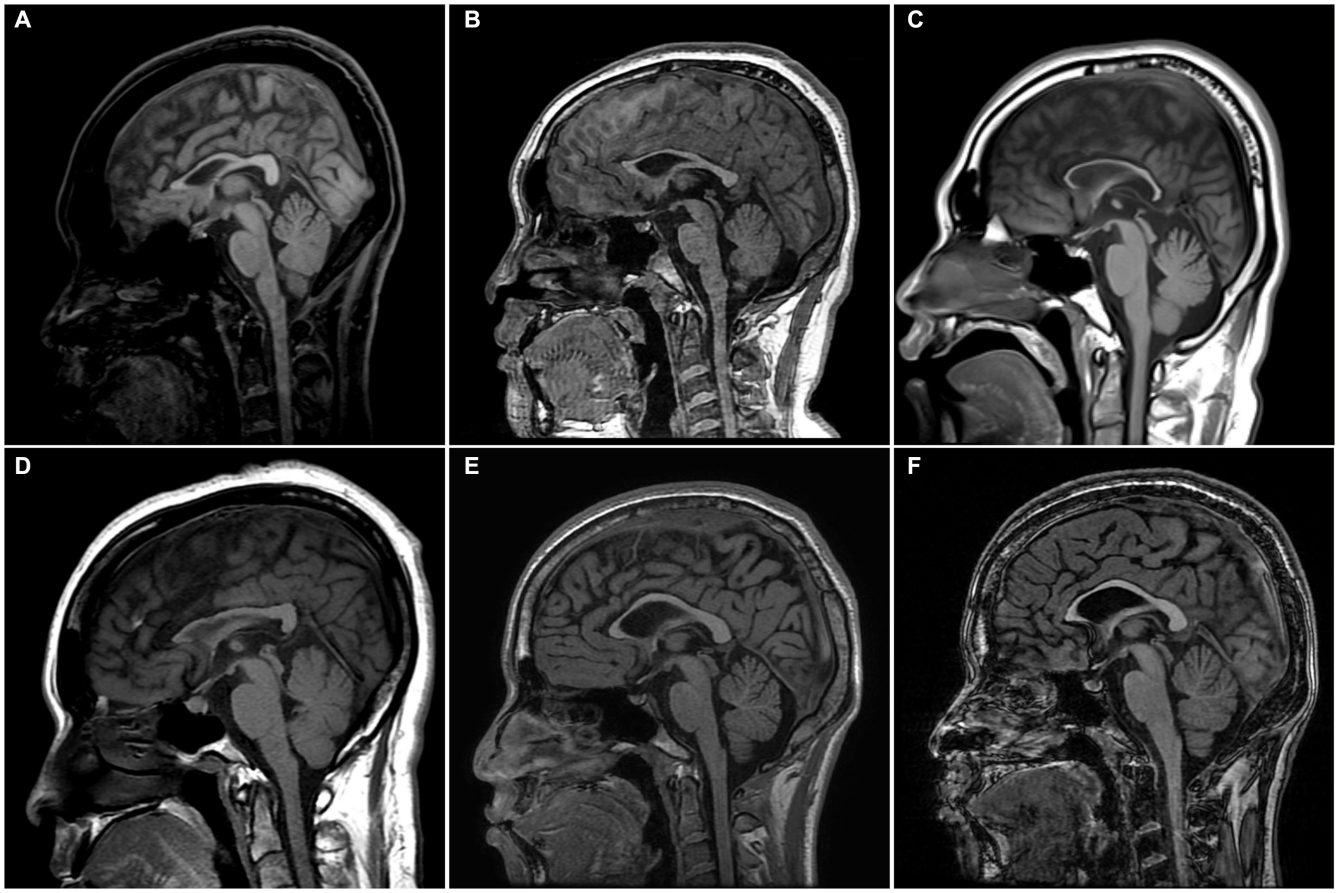
Sagittal T1-weight MR images of the enrolled patients. **(A)** SN-1, 18 years of age, has focal thinning of region 3 in the corpus callosum. **(B)** SN-2, 17 years of age, has thinning of regions 3 and 4. **(C)** SN-3, 22 years of age, has thinning of the entire corpus callosum involving regions 1–7. **(D)** SN-4, 41 years of age, has thinning of the anterior half of the corpus callosum involving regions 1–4. **(E,F)** SN-5 and SN-6, 21-year-old identical twins, have focal thinning of region 3. The corpus callosum was divided into seven regions according to Witelson’s guidelines. SN, subject number.

**Figure 2:**
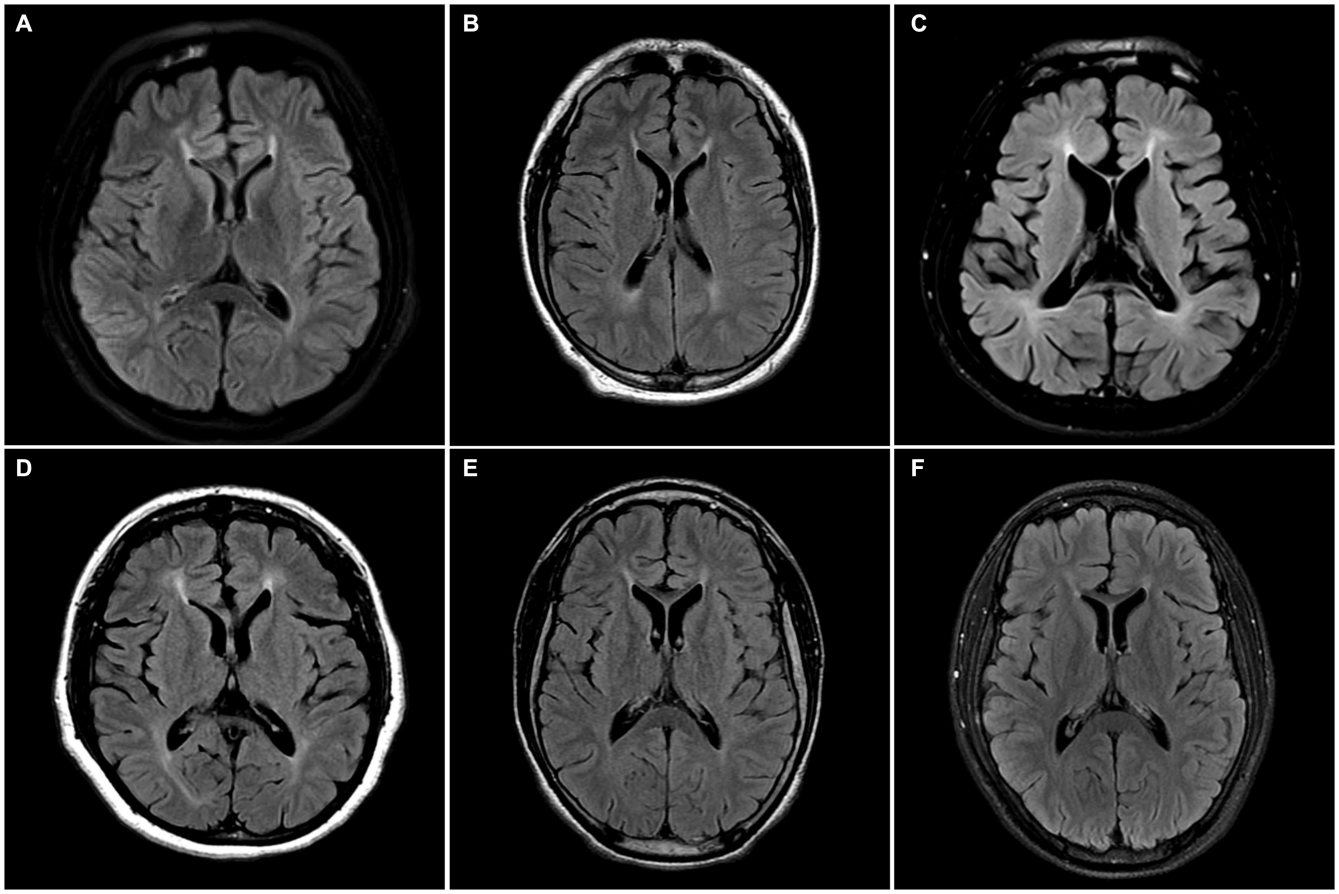
Axial FLAIR MR images of the enrolled patients. **(A–F)** All subjects have hyperintensities at the forceps minor region called the ears of the lynx sign. **(B–D)** SN-2, SN-3, and SN-4 have forceps major region signal abnormality. **(C)** SN-3 has the widening of the lateral ventricle.

**Figure 3:**
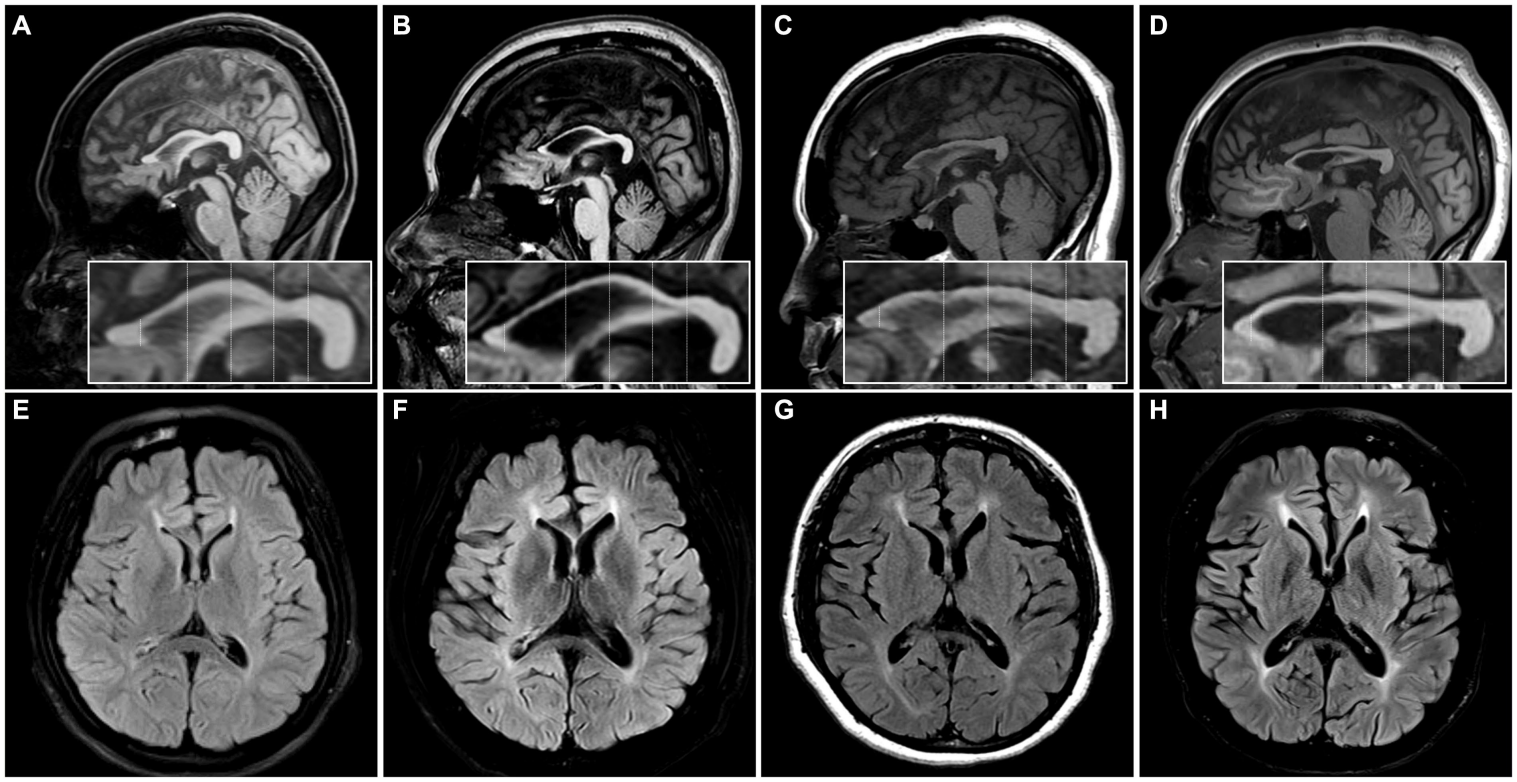
The initial and follow-up images of two patients. **(A–D)** MR images of SN-1. **(A,B)** The focal thinning of region 3 in the initial image was further thinned and other regions of the corpus callosum were also slenderized on the MRI performed 12 years later. **(C,D)** The forceps minor region signal abnormality became more distinct in the subsequent image, and the forceps major region signal abnormality with ventricular widening was observed in the follow-up image. **(E–H)** MR images of the SN-4. **(E,F)** The thinning in the anterior half of the corpus callosum was still observed on the MRI performed 3 years later, although there was no significant change. **(G,H)** The forceps minor and major regions signal abnormalities with ventricular widening were more distinct in the follow-up image.

Radiologically, TCC was observed in all subjects. Three subjects (SN-1, SN-5, and SN-6) showed focal thinning limited to region 3, and another (SN-2) showed thinning in regions 3 and 4. The other two (SN-3 and SN-4) showed diffuse thinning involving the entire or anterior half of the corpus callosum, respectively (Figure 1). The FLAIR hyperintensities at the forceps minor, called ears of the lynx sign, were found in all subjects, PVWM signal abnormalities at the forceps major were seen in three (SN-2, SN-3, and SN-4), and the widening of the lateral ventricles was found in one (SN-3; Figure 2). A follow-up brain MRI was performed after 12 years in one subject (SN-1) and 3 years in the other (SN-4). In SN-1, the thinning of region 3 had further progressed and the corpus callosum had become diffusely thinned. More pronounced FLAIR signal abnormalities at the forceps minor and major were accompanied by the enlargement of the lateral ventricles (Figures 3A–D). In SN-4, TCC did not significantly worsen, although PVWM signal abnormalities accompanied by ventricular widening became prominent (Figures 3E–H). The spine MRI performed on three subjects was unremarkable. In the electrodiagnostic studies, MEP to the lower limbs showed an absent response in all subjects, and MEP to the upper limbs was absent in three subjects (SN-1, SN-2, and SN-3). In the follow-up MEP tests performed 2 years later, upper limb CMCT was absent in one subject (SN-4) and prolonged in two subjects (SN-5 and SN-6). SEP revealed unremarkable results in both the upper and lower limbs. NCS was unremarkable, except for one subject (SN-4) who had distal symmetric axonal sensory polyneuropathies in the legs. Needle EMG showed myotonic discharges typical of non-dystrophic myotonia in the twins. Concerning neuropsychologic studies, the median K-MMSE score was 27 (range, 6–28), all of which were deducted in the serial sevens task. Cognitive ability was examined using WAIS-III in two (SN-1 and SN-2) or WAIS-IV in the others, and the median full-scale IQ was 48 (range, 42–76). The detailed information on the radiological, electrodiagnostic, and neuropsychologic findings is summarized in Table 3.

**Table 3:** Laboratory findings of the enrolled patients in this study.

Subjects	SN-1	SN-2	SN-3	SN-4	SN-5	SN-6
Radiologic study						
TCC	+	+	+	+	+	+
Involved region	R3	R3,R4	R1-R7	R1-R4	R3	R3
PVWM signal change	+	+	+	+	+	+
Forceps minor	+	+	+	+	+	+
Forceps major	−	+	+	+	−	−
Ventricular widening	− → +	−	+	− → +	−	−
Spinal cord	NL	NL	ND	NL	ND	ND
Electrodiagnostic study						
Initial MEP						
Lower limb CMCT	Absent	Absent	Absent	Absent	Absent	Absent
Upper limb CMCT	Absent	Absent	Absent	NL	NL	NL
Follow-up MEP						
Lower limb CMCT	Absent	Absent	Absent	Absent	Absent	Absent
Upper limb CMCT	Absent	Absent	Absent	Absent	Prolonged	Prolonged
SEP	NL	NL	NL	NL	NL	NL
Nerve conduction study						
Motor	NL	NL	NL	NL	NL	NL
Sensory	NL	NL	NL	Axonopathy	NL	NL
Neuropsychologic study						
Education (years)	11	9	7	16	9	9
K-MMSE (points)	27	26	26	28	27	27
Attention/calculation	2/5	1/5	1/5	3/5	2/5	2/5
FSIQ on K-WAIS	51^a^	42^b^	43	72	47	49
VCI	NA	NA	51	88	62	59
PRI	NA	NA	50	74	53	68
WMI	NA	NA	54	84	52	52
PSI	NA	NA	50	69	50	50

TCC, the thinning of the corpus callosum; PVWM, periventricular white matter; MEP, motor evoked potentials; CMCT, central motor conduction time; SEP, somatosensory evoked potentials; K-MMSE, the Korean version of the mini-mental state examination; K-WAIS, Korean Wechsler Adult Intelligence Scale; FSIQ, full-scale intellectual quotient; VCI, verbal comprehension index; PRI, perceptual reasoning index; WMI, working memory index; PSI, processing speed index; R, region; ND, not done; NL, normal; NA, not available.

a K-WAIS-III in SN-III revealed 57 of verbal IQ, and 53 of performance IQ.

b K-WAIS-III in SN-IV revealed 44 of verbal IQ and 40 of performance IQ.

## Discussion

In addition to spastic weakness in the legs, a core feature of HSP, the major additional symptoms of SPG11-HSP were learning difficulties, pseudobulbar dysarthria, bladder dysfunction, and being overweight. A few subjects complained of rigidity in their upper limbs or neuropathic pain, which are known as possible accompanying manifestations (4, 5, 17, 18). The twins presented myotonic muscle stiffness, which has been reported in a family with autosomal recessive HSP (19). However, it could be due to an incidental coexistence of myotonia congenita rather than an additional symptom of SPG11-HSP, given that a pathogenic *CLCN1* mutation was identified. Intriguingly, all subjects had abnormal BMI scores of 25 kg/m^2^ or higher, indicating they were overweight according to the WHO classification, yet implying obesity when applying the refined cut-off for Asian-Pacific populations (20, 21). Although metabolic aspects have not been well implicated in SPG11-HSP, recent studies have suggested a relationship between bodyweight changes and hypothalamic dysfunction in this disease (22, 23). Therefore, weight gain may not be simply because of physical inactivity following gait disturbance but an additional manifestation of SPG11-HSP (22). Furthermore, since body composition and the related metabolic profile of the Asian population can differ from Western society (24), further investigations into changes in bodyweight among SPG11-HSP patients by race may be warranted.

The distinct radiologic finding in this study was TCC. We divided the corpus callosum into subregions to identify the thinning pattern since the callosal fibers of the corpus callosum are thought to have a topographic organization in accordance with projecting cortical areas (25, 26). The most commonly affected area was region 3 or 4, which contains commissural fibers to the premotor and motor cortex (12, 27). Considering that a patient’s serial MRI images spaced over 10 years showed aggravation of TCC, the thinning may extend from the rostral or anterior midbody to the entirety of the corpus callosum with disease progression (5, 18, 28, 29). In addition, all subjects had PVWM signal abnormalities in common, even those who had mild and focal TCC. Notably, these signal changes worsened without further thinning of the corpus callosum on the follow-up MRI of SN-4. Thus, PVWM signal abnormality, including the ears of the lynx sign, seemed to parallel or precede the worsening of TCC (30). The enlargement of lateral ventricles observed in three subjects has been rarely described before (31, 32), and is presumed to be related to the decreased white and grey matter volume (31).

Peripheral neuropathy has been infrequently reported in SPG11-HSP (5). Its clinical symptoms are usually mild, but it has been reported that severe axonopathy, neuropathic pain, or autonomic dysfunction may be accompanied in the late stage of the disease (17). In this study, NCS was abnormal in the only patient with neuropathic pain, which is inconsistent with a large cohort study of 25 SPG11-HSP subjects, in which 96% subjects showed non-length dependent motor neuropathy (33). Interestingly, all subjects showed absent lower limb CMCT regardless of SPRS scores or disease duration, similar to the many studies on the neurophysiologic features of HSP that have reported abnormal lower limb MEP (34). Meanwhile, less attention has been paid to MEP in the upper limbs, and investigations specific to SPG11-HSP are still lacking, even though it is the most common subtype of autosomal recessive HSP. In this study, the frequency of abnormal findings in the upper limb MEP was relatively high compared to an abnormal rate of 26% in a systemic review covering HSP patients with variable genotypes (34). MEP is thought to reflect the neuronal integrity of the upper motor neurons and has been proposed as a disease severity marker (34–36). Therefore, rapid conversion of the upper limb CMCT abnormalities may reflect progressive neurodegeneration of SPG11-HSP, and its longitudinal evaluation of the MEP test could provide a clue to assess the clinical course of this disease.

Intellectual disability is known as one of the non-motor symptoms of SPG11-HSP (37). The MMSE scores of the subjects were slightly lower for age and education, which is in line with the fact that intellectual disability is a frequent feature of SPG11-HSP (38). The MMSE is a 30-point questionnaire composed of several domains, although few papers on HSP have described it in detail by domain. Notably, all subjects exclusively showed impairment in the attention/calculation domain, which evaluates working memory. Contrarily, IQ was conspicuously lower than the age-matched population in five patients, and one showed borderline intellectual functioning. A similar discrepancy between the Montreal Cognitive Assessment and MMSE was observed in a study of four patients with SPG11-HSP (39). Thus, additional comprehensive assessments of intellectual function would be needed, even if the MMSE score seems satisfactory. Nonetheless, since the MMSE is the most widely used cognitive screening tool in clinical settings, the serial sevens task may be a straightforward and useful method to identify cognitive impairment in those suspected of complicated HSP.

This study has several limitations. First, small number of patients were studied. Second, this study was designed retrospectively. Third, copy number variants are known to account for about 19% of pathogenic *SPG11* alleles (40), but no further genetic tests, such as multiplex ligation-dependent probe amplification and segregation analysis, were performed. Nevertheless, the subjects had clinicoradiological features consistent with SPG11-HSP, and they only carried one or two pathogenic variants in the *SPG11* gene without clinically or genetically significant variants in other HSP-related genes. Additional prospective studies with larger sample sizes are necessary to comprehensively understand the clinical features of patients with SPG11-HSP.

## Data availability statement

The original contributions presented in the study are included in the article/Supplementary material, further inquiries can be directed to the corresponding author.

## Ethics statement

The studies involving human participants were reviewed and approved by Institutional Review Board at Chonnam National University Hospital. The patients/participants provided their written informed consent to participate in this study.

## Author contributions

Y-RK: data curation and writing-original draft. T-SN: conceptualization, writing-review and editing, and supervision. J-MK, KK, and BK: data curation and formal analysis. S-MC and S-HL: formal analysis. M-KK: formal analysis and supervision. All authors contributed to the article and approved the submitted version.

## Funding

This study was supported by the Health Fellowship Foundation and a grant from the Chonnam National University Hospital Biomedical Research Institute (BCRI 21025).

## Conflict of interest

The authors declare that the research was conducted in the absence of any commercial or financial relationships that could be construed as a potential conflict of interest.

## Publisher’s note

All claims expressed in this article are solely those of the authors and do not necessarily represent those of their affiliated organizations, or those of the publisher, the editors and the reviewers. Any product that may be evaluated in this article, or claim that may be made by its manufacturer, is not guaranteed or endorsed by the publisher.
