# Supplementary material for: Clinical analysis in patients with SPG11 hereditary spastic paraplegia
Source: Front Neurol. 2023 Jun 15;14:1198728. doi: 10.3389/fneur.2023.1198728 (PMC10310533; doi:10.3389/fneur.2023.1198728)
Supplement: Supplementary file 1 [file Data_Sheet_1.DOCX]

**Supplementary Method: Whole exome sequencing analysis**

This study utilized The Agilent SureSelect Target Enrichment protocol for Illumina paired-end sequencing libraries to generate standard exome capture libraries. The SureSelect Human All Exon V6 probe set was used. The quantification and quality of the DNA were measured by PicoGreen and agarose gel electrophoresis. An amount of 1 μg of genomic DNA from each cell line was diluted in EB buffer and sheared to a target peak size of 150–200 bp using the Covaris LE220 focused-ultrasonicator (Covaris, Woburn, MA), according to the manufacturer's instructions. The 8 microTUBE Strip was loaded into the tube holder of the ultrasonicator, and the DNA was sheared using the following settings: mode: frequency sweeping; duty cycle: 10%; intensity: 5; cycles per burst: 200; duration: 60 sec for 6 cycles; temperature: 4 °C–7 °C. The fragmented DNA was repaired with A-tailing at the 3′ ends, and Agilent adapters were ligated to the fragments. After assessing ligation, the adapter-ligated product was polymerase chain reaction (PCR) amplified. The final purified product was quantified using the TapeStation DNA screentape D1000 (Agilent). For exome capture, 250 ng of the DNA library was mixed with hybridization buffers, blocking mixes, RNase block, and 5 µl of SureSelect all exon capture library. Hybridization to the capture baits was conducted at 65 °C using the heated thermal cycler lid option at 105 °C for 24 hours on a PCR machine. Then, the captured DNA was washed and amplified. Then, the final purified product was quantified using qPCR, according to the qPCR Quantification Protocol Guide Guide (KAPA Library Quantification kits for Illumina Sequencing platforms), and qualified using the TapeStation DNA screentape D1000 (Agilent). Next, the Indexed libraries were sequenced using the NovaSeq6000 platform (Illumina, San Diego, USA).

Paired-end sequences were first mapped to the human genome, without unordered sequences and alternate haplotypes, using the mapping program ‘BWA’ (version 0.7.12). The reference sequence was UCSC assembly hg19 (original GRCh37 from NCBI, Feb. 2009). A mapping result file in BAM format was generated using ‘BWA-MEM.’ Then, programs packaged in Picard tools (ver.1.130) were applied to remove PCR duplicates, reducing identically matching reads at the start position into a single read using MarkDuplicates.jar. Base quality score recalibration (BQSR) and local realignment around InDels were performed using the Genome Analysis Toolkit (GATK3.4), such that the number of mismatching bases was minimized across all the reads. Variant genotyping for each sample was performed with HaplotypeCaller of GATK (GATKv3.4.0). Based on the previously generated BAM file, the variant genotyping for each sample was performed with the GATK HaplotypeCaller. In this stage, SNP and short InDels candidates were detected at the nucleotide resolution. Those variants were annotated to VCF file format by another program, SnpEff v4.1g, filtering with dbSNP for the version of 142 and SNPs from the 1000 genome project. Then, an in-house program and SnpEff were applied to filter additional databases, including ESP6500, ClinVar, KRGDB (the Korean Reference Genome Database: 1100 Koreans), and dbNSFP2.9.

The discovered variants were classified and annotated using the Golden Helix VarSeq analysis workflow, which implements the American College of Medical Genetics and Genomics guideline. The following databases and in silico algorithms were used to annotate and evaluate the impact of the variant in the context of human disease: 1000 genomes, gnomAD, ClinVar, OMIM, dbSNP, NCIB RefSeq Genes, ExAC Gene Constraints, VS-SIFT, VS-PolyPhen2, PhyloP, GERP++, GeneSplicer, MaxEntScan, NNSplice, and PWM Splice Predictor. Analysis was reported with the HGVS nomenclature implemented by the VarSeq transcript annotation algorithm.
