# Supplementary figures and images for: Clinical analysis in patients with SPG11 hereditary spastic paraplegia
Source: Front Neurol. 2023 Jun 15;14:1198728. doi: 10.3389/fneur.2023.1198728 (PMC10310533; doi:10.3389/fneur.2023.1198728)

**Supplementary Figure 1. Study population selection**

**
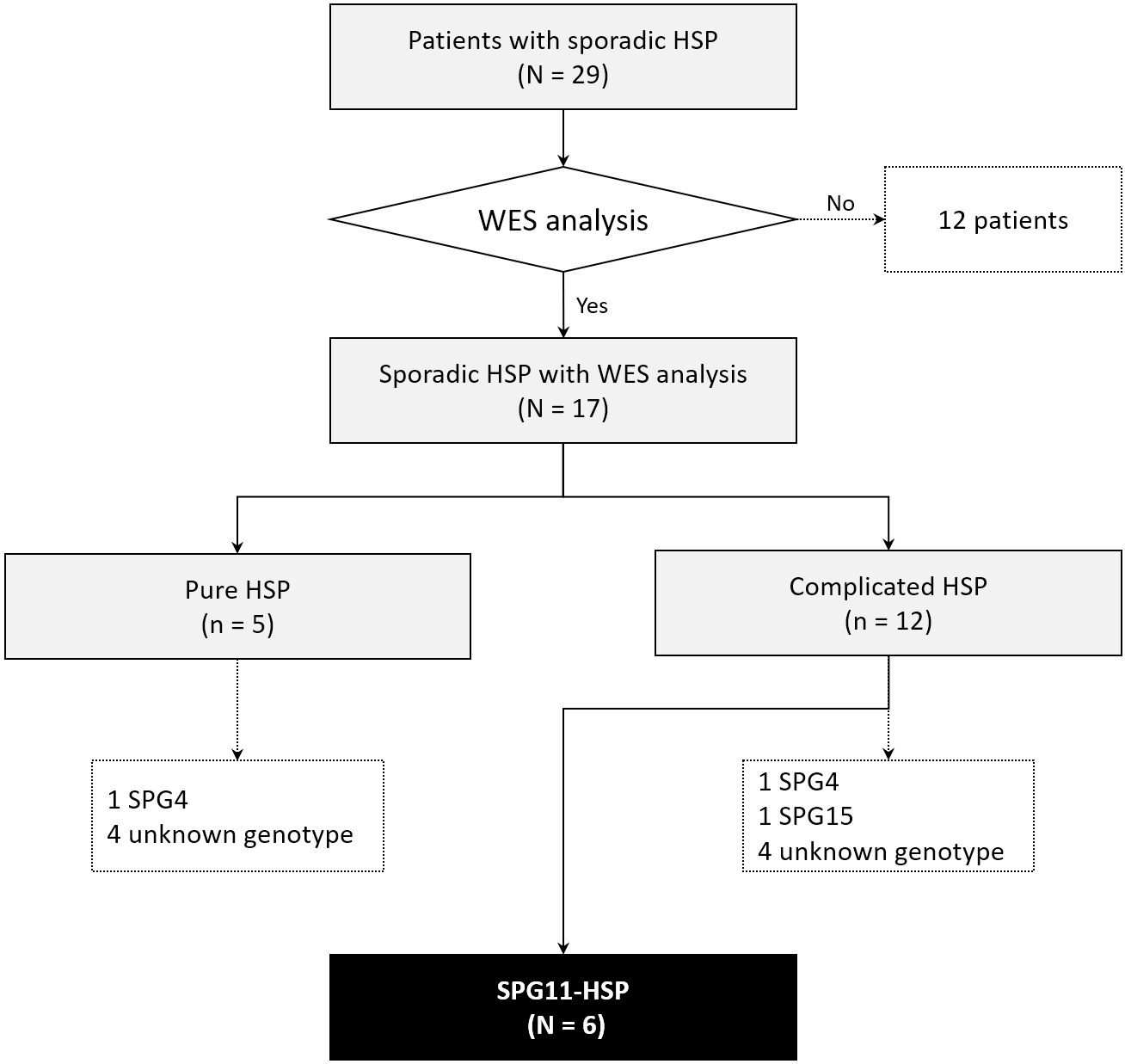
**

Supplement: Supplementary file 2 [file Data_Sheet_2.docx]
