# Supplementary material for: Clinical analysis in patients with SPG11 hereditary spastic paraplegia
Source: Front Neurol. 2023 Jun 15;14:1198728. doi: 10.3389/fneur.2023.1198728 (PMC10310533; doi:10.3389/fneur.2023.1198728)
Supplement: Supplementary file 3 [file Table_1.DOCX]

| **Supplementary Table 1.** **Variants found in the SPG11 gene through WES analysis** | | | | | |  |
| --- | --- | --- | --- | --- | --- | --- |
| **Patient** | **c.DNA Change** | **Zygosity** | **Expect on protein** | **dbSNP No.** | **Reference** |  |
| SN-1 | c.667+103T>C | Heterozygous | NA | rs7165146 |  |  |
|  | c.1388T>C | Homozygous | p.Phe463Ser | rs3759871 |  |  |
|  | c.3146-102G>T | Homozygous | NA | rs12909507 |  |  |
|  | **c.3291+1G>T** | **Heterozygous** | **NA** | **rs312262753** | 1 |  |
|  | **c.4307_4308delAA** | **Heterozygous** | **p.Gln1436Argfs** | [**rs312262759**](https://www.ncbi.nlm.nih.gov/snp/rs312262759) | 2-4 |  |
|  | c.5121+33delT | Homozygous | NA | NA |  |  |
| SN-2 | c.257+35G>A | Heterozygous | NA | rs117101120 |  |  |
|  | c.442+99C>T | Heterozygous | NA | rs75927439 |  |  |
|  | c.667+103T>C | Heterozygous | NA | rs7165146 |  |  |
|  | c.1008-87T>C | Heterozygous | NA | rs78975199 |  |  |
|  | c.1347C>T | Heterozygous | p.Thr449Thr | rs3759874 |  |  |
|  | c.1348A>G | Heterozygous | p.Ile450Val | rs3759873 |  |  |
|  | c.1603-46_1603-45insT | Heterozygous | NA | rs199611470 |  |  |
|  | c.2621-153_2621-150delAAAA | Homozygous | NA | rs137921465 |  |  |
|  | c.3146-102G>T | Homozygous | NA | rs12909507 |  |  |
|  | **c.3291+1G>T** | **Heterozygous** | **NA** | **rs312262753** | 1 |  |
|  | c.3520+2018G>A | Heterozygous | NA | rs78119351 |  |  |
|  | c.6330G>A | Heterozygous | p.Gly2110Gly | rs35932349 |  |  |
|  | c.6344-74C>T | Heterozygous | NA | rs2277610 |  |  |
|  | c.6755-17_6755-15delCTT | Heterozygous | NA | rs143026515 |  |  |
| SN-3 | **c.200_203delCTTT** | **Heterozygous** | **p.Ser67fs** | **NA** |  |  |
|  | c.257+35G>A | Heterozygous | NA | rs117101120 |  |  |
|  | c.667+103T>C | Heterozygous | NA | rs7165146 |  |  |
|  | c.1008-87T>C | Heterozygous | NA | rs78975199 |  |  |
|  | c.1347C>T | Heterozygous | p.Thr449Thr | rs3759874 |  |  |
|  | c.1348A>G | Heterozygous | p.Ile450Val | rs3759873 |  |  |
|  | c.1603-46_1603-45insT | Homozygous | NA | rs199611470 |  |  |
|  | c.3520+2018G>A | Heterozygous | NA | rs78119351 |  |  |
|  | c.4161+118_4161+123delTTTTTT | Homozygous | NA | NA |  |  |
|  | c.6330G>A | Heterozygous | p.Gly2110Gly | rs35932349 |  |  |
|  | c.6344-74C>T | Heterozygous | NA | rs2277610 |  |  |
|  | c.6755-17_6755-15delCTT | Heterozygous | NA | rs143026515 |  |  |
|  | **c.7010T>G** | **Heterozygous** | **p.Val2337Gly** | **NA** |  |  |
| SN-4 | c.667+103T>C | Homozygous | NA | rs7165146 |  |  |
|  | c.1388T>C | Homozygous | p.Phe463Ser | rs3759871 |  |  |
|  | **c.2987_2989delGTT** | **Heterozygous** | **p.Cys996del** | **NA** | 5,6 |  |
|  | c.3146-102G>T | Homozygous | NA | rs12909507 |  |  |
|  | c.3146-6T>G | Heterozygous | NA | NA |  |  |
|  | c.5121+33delT | Homozygous | NA | NA |  |  |
|  | c.6754+74_6754+75insT | Heterozygous | NA | NA |  |  |
| SN-5/6 | c.667+103T>C | Homozygous | NA | rs7165146 |  |  |
|  | c.833A>G | Heterozygous | p.Asn278Ser | rs75309308 |  |  |
|  | c.993C>T | Heterozygous | p.Ser331Ser | rs76823973 |  |  |
|  | c.1008-87T>C | Heterozygous | NA | rs78975199 |  |  |
|  | c.1388T>C | Heterozygous | p.Phe463Ser | rs3759871 |  |  |
|  | **c.2163dupT** | **Heterozygous** | **p.Ile722Tyrfs** | [**rs312262738**](https://www.ncbi.nlm.nih.gov/snp/rs312262738) | 7 |  |
|  | c.3146-102G>T | Homozygous | NA | rs12909507 |  |  |
|  | c.3520+2018G>A | Heterozygous | NA | rs78119351 |  |  |
|  | **c.5410_5411delTG** | **Heterozygous** | **p.Cys1804Profs** | [**rs312262766**](https://www.ncbi.nlm.nih.gov/snp/rs312262766) | 1 |  |
|  | c.6754+74delT | Heterozygous | NA | rs397853802 |  |  |
|  | c.6844-71G>A | Heterozygous | NA | rs74363814 |  |  |
| SN-5 and SN-6 are identical twins. The variants described in Table 1 are shown in bold. NA: not available. | | | | | | |

**References for the Supplementary Table 1**

1. Kim SM, Lee JS, Kim S, et al. Novel compound heterozygous mutations of the SPG11 gene in Korean families with hereditary spastic paraplegia with thin corpus callosum. J Neurol 2009;256:1714-1718.
2. Zhang SS, Chen Q, Chen XP, et al. Two novel mutations in the SPG11 gene causing hereditary spastic paraplegia associated with thin corpus callosum. Mov Disord 2008;23:917-919.
3. Denora PS, Schlesinger D, Casali C, et al. Screening of ARHSP-TCC patients expands the spectrum of SPG11 mutations and includes a large scale gene deletion. Hum Mutat 2009;30:E500-519.
4. Stevanin G, Azzedine H, Denora P, et al. Mutations in SPG11 are frequent in autosomal recessive spastic paraplegia with thin corpus callosum, cognitive decline and lower motor neuron degeneration. Brain 2008;131(Pt 3):772-784.
5. Elert-Dobkowska E, Stepniak I, Krysa W, et al. Next-generation sequencing study reveals the broader variant spectrum of hereditary spastic paraplegia and related phenotypes. Neurogenetics 2019;20:27-38.
6. Günther S, Elert-Dobkowska E, Soehn AS, et al. High Frequency of Pathogenic Rearrangements in SPG11 and Extensive Contribution of Mutational Hotspots and Founder Alleles. Hum Mutat 2016;37:703-709.
7. Liao SS, Shen L, Du J, et al. Novel mutations of the SPG11 gene in hereditary spastic paraplegia with thin corpus callosum. J Neurol Sci 2008;275:92-99.
